# Should the Health Community Promote Smokeless Tobacco (Snus): Author's Reply

**DOI:** 10.1371/journal.pmed.0040299

**Published:** 2007-10-30

**Authors:** Simon Chapman

As someone who has researched and advocated for harm reduction in the HIV/AIDS [1] and narcotics areas [2], I am highly supportive of the general principle of reducing harm in public health. My recently released book [3] features a 29,000 word chapter examining the application of the term in tobacco control. My current position is that there is an overly seductive simplicity in drawing neat analogies with other areas of harm reduction when it comes to tobacco. It is obvious that there is immediate benefit to health and society from encouraging condom use and clean needle use. However, the putative benefits of population experiments with harm reduction will not be assessable for 30–40 years.

Behind most calls for harm reduction in tobacco control policy lie under-examined assumptions that there is a large intractable smoking population for whom cessation is “an impossible goal,” as Maggie Brown puts it [4]. In New South Wales, Australia where I live, only 13.9% of people aged 14 and over now smoke daily. In recent years smoking prevalence has been falling faster than at any time in the past. There is poor evidence for the “hardening” hypothesis, with 29% of smokers now describing themselves as only occasional smokers and daily consumption falling [5], facts incompatible with hardening. Around 75% of smokers say they wish to quit, and only about 3.5% of smokers say they want to continue using tobacco. It is a fraction of this group that harm reduction advocates seek to interest.

While a case may exist for carefully controlled access to snus by such a relatively small group, the case for allowing the foxes in the tobacco industry into the chicken coop of open sales and marketing should alarm anyone with their eyes open to the industry's bottom line. Recent insights confirm our caution that Big Tobacco sees snus as a way of arresting declines in smoking by promoting dual use. Citigroup, the investment advisors, are very clear on the way snus will be marketed and used, writing “Over 60% of our survey respondents [in the tobacco trade] do not believe snus products will have an impact on cig volumes. The trade believes that snus will be consumed in addition to cigarettes. Given the increased bans on smoking, snus products seem like an obvious substitution” [6]. This was echoed in the US retail trade newsletter Brandweek: “There's money to be made from municipal smoking bans as another cigarette maker chases after smokers who get their nicotine fix between their cheek and gum during those many moments when they can't light up” [7].

I'm certain that your British American Tobacco correspondents [8] “understand” that cigarettes cause disease, as they put it. That being the case, might we anticipate British American Tobacco planning to end sales of its cigarettes in the areas in which it is test marketing same-name brand snus? Does it plan to run aggressive, effective, graphic advertising campaigns on the dangers of smoking its cigarettes?
